# Cytotoxic effects of *Mangifera indica* L. kernel extract on human breast cancer (MCF-7 and MDA-MB-231 cell lines) and bioactive constituents in the crude extract

**DOI:** 10.1186/1472-6882-14-199

**Published:** 2014-06-25

**Authors:** Al-Shwyeh Hussah Abdullah, Abdulkarim Sabo Mohammed, Rasedee Abdullah, Mohamed Elwathig Saeed Mirghani, Mothanna Al-Qubaisi

**Affiliations:** 1 Faculty of Food Science and Technology, Universiti Putra Malaysia, 43400 Serdang, Selangor, Malaysia; 2 Faculty of Veterinary Medicine, Universiti Putra Malaysia, 43400 Serdang, Selangor, Malaysia; 3 Institute of Bioscience, Universiti Putra Malaysia, 43400 Serdang, Selangor, Malaysia; 4 Department of Biotechnology Engineering Faculty of Engineering, International Islamic, University Malaysia (IIUM), P.O. BOX 10, 50728 Kuala Lumpur, Malaysia; 5 Department of Cell and Molecular Biology, Faculty of Biotechnology, Universiti Putra, Malaysia, 43400 Serdang, Selangor, Malaysia

**Keywords:** *Mangifera indica* L, kernel extract, MCF-7 cells, MDA-MB-231 cell, Cytotoxicity, Anticancer activity

## Abstract

**Background:**

Waterlily Mango (*Mangifera indica* L.) is thought to be antioxidant-rich, conferred by its functional phytochemicals.

**Methods:**

The potential anticancer effects of the ethanolic kernel extract on breast cancer cells (MDA-MB-231 and MCF-7) using MTT, anti-proliferation, neutral red (NR) uptake and lactate dehydrogenase (LDH) release assays were evaluated. Cytological studies on the breast cancer cells were also conducted, and phytochemical analyses of the extract were carried out to determine the likely bioactive compounds responsible for such effects.

**Results:**

Results showed the extract induced cytotoxicity in MDA-MB-231 cells and MCF-7 cells with IC_50_ values of 30 and 15 μg/mL, respectively. The extract showed significant toxicity towards both cell lines, with low toxicity to normal breast cells (MCF-10A). The cytotoxic effects on the cells were further confirmed by the NR uptake, antiproliferative and LDH release assays. Bioactive analyses revealed that many bioactives were present in the extract although butylated hydroxytoluene, a potent antioxidant, was the most abundant with 44.65%.

**Conclusions:**

*M. indica* extract appears to be more cytoxic to both estrogen positive and negative breast cancer cell lines than to normal breast cells. Synergistic effects of its antioxidant bioactives could have contributed to the cytotoxic effects of the extract. The extract of *M. indica*, therefore, has potential anticancer activity against breast cancer cells. This potential is worth studying further, and could have implications on future studies and eventually management of human breast cancers.

## Background

Breast cancer causes significant morbidity and mortality among women [1], and metastasis mainly affects outcome of the disease [2]. Lack of effective therapeutic strategies for control and treatment of breast cancers, and the huge financial burden placed on individuals and nations mean urgent action must be taken in the fight against breast cancer. Also, side effects due to conventional pharmacological agents have necessitated the search for newer therapies mostly in the form of natural products. In recent years, interest in natural products has grown, and in the light of long-term and safe cancer prevention, current approaches have been focused on the use of food and edible medicinal herbs as sources of products that could effectively control cancers [3-5]. This is evident by the fact that approximately 74% of new anticancer compounds are either natural products or natural product-derived [6-9]. In fact, it has been argued that plants may be sources of multiple bioactive compounds that could provide more benefit than single pharmacological agents against chronic diseases, and this may well be beneficical in managing breast cancer. The antioxidant potentials of plant bioresources, mainly contributed by their bioactive compounds, have been closely linked to their abilities to suppress growth of cancer cells, likely through reduced oxidative stress, which may play a role in the development and progression of cellular damages underlying cancerous growth. As such, it has been suggested that antioxidant supplementation may reduce breast cancer recurrence and mortalities [10] and through bioassay systems and animal studies, there have been indications that numerous naturally-occurring antioxidant compounds possess anticancer properties [11,12]. In particular, consumption of foods and beverages rich in polyphenols such as catechins, antocyanines and flavones have been linked to lower occurrence of cancers [13]. Other phenolic compounds claimed to possess biological activities include coumarins, lignans, phenolic acids, flavonoids, quinones, stilbenes, tannins and curcuminoids [14].

*Mangifera indica* L. is a popular fruit crop in tropical and subtropical areas of the world, specifically Asia, because of its characteristic taste, colour and nutritional value [15,16]. It has numerous classes of bioactive compounds and vitamins with different health-promoting characteristics [17]. The ethanolic extract of its peel was observed to have great antioxidant and anti-proliferative properties, attributed to its phenolic content [18,19]. Notwithstanding these reports however, the phytochemical profile or therapeutic potential of compounds in the kernel has not been studied. Thus, the phytochemical profile and the potential cytotoxic effects of the kernel extract towards breast cancer cell lines were studied and evaluated.

## Methods

### Chemicals and reagents

Dulbecco’s modified Eagle medium (DMEM), thiazoyl blue tetrazolium bromide (MTT), 95% ethanol, dimethylsulphoxide (DMSO), nicotinamide adenine dinucleotide (reduced form) NADH, fetal bovine serum (FBS), phosphate buffer saline (PBS), trypan blue dye solution, trypsin-EDTA, neutral red (NR) solution and antibiotics were purchased from Sigma-Aldrich (St. Louis, MO, USA).

### Raw materials

Waterlily mango (*Mangifera indica* L.) fruits were procured from a local market in Kuala Lumpur, Malaysia and were identified by a resident botanist and voucher specimen (Mr. Shamsul Khamis) from Institute of Bioscience, Universiti Putra Malaysia under the voucher specimen (SK2448/14).The mango kernel was manually isolated from the stone and flesh.

### Preparation of crude extract

*M. indica* kernels were soaked in water, and washed to remove adhering flesh. They were then air-dried, and subsequently kept in an oven at 45°C for 2 days. The dried kernels were finely ground with a Waring blender 7011HS (Osaka Chemical Co. Ltd., Osaka, Japan) and stored at 4°C until analysis. Ethanol (95%) was added to the kernel powder at 10:1 (v/w) and the mixture shaken continuously at 200 rpm and 37°C for 24 h in an incubator shaker (INNOVA 4000, New Jersey, USA). Insoluble materials were then removed by filtration and the filtrates centrifuged for 10 min at 4000 rpm using Benchtop Centrifuge Z200A (Labnet International, Inc., Woodbridge, NJ, USA). The residues were discarded and the supernatant dried using 1 L Rotary Evaporator N1001S-WD (Tokyo Rikakikai Co., Ltd., Tokyo, Japan) until the extract was fully concentrated. After determining the yield, the concentrated extract was then dissolved in DMSO and stored in a freezer at -20°C [20] before further analyses.

### In vitro assay

Two human breast cancer cell lines, MDA-MB-231 and MCF-7 cells and one normal cell line, MCF-10A cells were obtained from the American Type Culture Collection (ATCC: Rockville, MD, USA). Cells were cultured in DMEM supplemented with 10% FBS and 1% antibiotics (100 U/ml penicillin) in an incubator at 37°C with 5% CO_2_.

### MTT assay

MCF-7, MDA-MB-231 and MCF-10A cells were seeded into 96-well plates at densities of 2 × 10^3^/well under 5% CO_2_ at 37°C for 24 h as reported previously [21]. The cells were then either treated with different concentrations of ethanolic extract of the *M. indica* kernel (10-1000 μg/mL) or doxorubicin (0.1-10 μg/mL) as positive control. After 72 h incubation, 20 μL of MTT solution (5 mg/mL) was pipetted into each well and incubated for another 4 h. The medium was later discarded and the formazan precipitate was dissolved in DMSO. The absorbance of the mixtures was determined using a microtiter plate reader at 570 and 630 nm (background) and the cell viability expressed as percentage of live cells relative to controls. All experiments were performed in triplicates. The IC_50_ was generated from the dose–response curve for each cell line.

### Anti-proliferation assay

MCF-7 and MDA-MB-231 cells were seeded into 6-well plates at densities of 1 × 10^4^ cells/well and allowed to incubate for 24 h for cell attachment. These exponentially growing cells were then exposed to 5, 10 and 50 μg/mL concentrations of the extract and the plates incubated at 37°C under 5% CO_2_, for 24, 48, and 72 h. At the end of the incubation periods, the medium was aspirated off and washed with cold PBS followed by the addition of 1 mL of 0.05% trypsin-EDTA. The plates were then incubated at 37°C for 15 min and after majority of the cells had detached from the plate, they were harvested by spinning the suspension for 10 min at 1000 rpm using Benchtop Centrifuge Z200A (Labnet International, Inc., Woodbridge, NJ, USA) and the supernatant discarded. Twenty microliters of the cell pellet were re-suspended in 20 μL of 0.4% trypan blue solution. The dye-excluding viable cells were counted microscopically using a hemocytometer, and expressed as percent of control cells that were still viable.

### Neutral red uptake assay

The cells were seeded in 96-well plates and incubated overnight at 37°C under 5% CO_2_ until they reached 60% confluence. The medium was then discarded and replaced with 200 μL of fresh growth medium containing the same concentrations of the kernel extract as that used in the MTT assay. Untreated cells under the same conditions were used as controls. The plates were incubated at 37°C under 5% CO_2_ for 24, 48 and 72 h and the cells were then washed three times with 200 μL of PBS. The plates were re-incubated for 3 h at 25°C in medium containing 200 μL NR solutions, and the cells subsequently washed to remove the NR solution. Cells were then exposed to fixing solution consisting of 1% CaCl_2_ and 0.5% formaldehyde in milli-Q water for 2 min followed by two washes with 1% acetic acid and 50% ethanol in milli-Q water. After a second wash, the plates were incubated for 10 min and then read in a microplate reader at 540 nm.

### Lactate dehydrogenase release assay

The permeability of the cell membrane of MCF-7 and MDA-MB-231 cell lines after treatment with ethanolic kernel extract was determined by LDH release assay. The cells were seeded in 96-well plates in 100 μL of media, and then treated with different concentrations of ethanolic extract of the *M. indica* kernel (10–1000 μg/mL) or doxorubicin (0.1-100 μg/mL). The treated cells then were incubated for 18 h, after which 40 μL of the medium was transferred to a new 96-well plate and further incubated for 72 h to determine LDH release. Forty microliter of 6% triton X-100 was added to the original 96-well plates to determine the total LDH concentration. An aliquot of 100 μL of 4.6 mM pyruvic acid in 0.1 M potassium phosphate buffer (pH 7.5) was added to each well of the plate containing the medium followed by 100 μL of 0.4 mg/mL reduced β-NADH in 0.1 M potassium phosphate buffer (pH 7.5). The kinetic change in absorbance at 340 nm was read for 1 min in an ELISA microplate reader. Change in 0.001 absorbance unit/min was considered to be equivalent to 1 U/L of LDH activity [21]. To determine total LDH activity, the procedure was repeated with 40 μL total cell lysate from untreated controls following similar way as stated above. The LDH release was determined as percentage of LDH in medium in comparison to total LDH in cell lysate of each respective well using the following equation: 100%* LDH_out_/(LDH_out_ + LDH_in_). Values are expressed as mean ± std. dev. (*n* = 3).

### Bioactive compounds analysis

The crude ethanolic extract of Waterlily kernel (100 mg) was dissolved in 10 mL of hexane and 100 μL of 2 N potassium hydroxide in methanol was added to the mixture and vortexed for 30 sec. The solution was further separated by centrifugation at 10,000 rpm for 10 minutes and the supernatant (1.5 mL) was transferred into a vial and analyzed using Gas chromatography–mass spectrometry following the method of Hema et al. [22]. The analysis of the extract was performed using a Perkin-Elmer GC claurus 500 system. The gas chromatograph was interfaced to a mass spectrometer, equipped with Elite-1 fused silica capillary column (30 m × 0.25 mm ID × 1 μm df, composed of 100% Dimethyl poly siloxane). Compounds were detected using an electron ionization system with ionization energy of 70 eV. Helium gas (99.999%) was used as the carrier gas at a constant flow rate of 1 mL/min and an injection volume of 0.5 μL, with a split ratio of 10:1. The injector temperature was 250°C while that of the ion source was 280°C. The oven temperature was set at 110°C held for 2 min with an increase of 10°C/min to 200°C, then at 5°C/min to 280°C held for 9 min. Mass spectra were taken at 70 eV; a scan interval of 0.5 sec and fragments from 45 to 450 Da. Total GC running time was 60 min.

Interpretation of mass spectrum from GC-MS was conducted using the database of National Institute of Standards and Technology (NIST). The spectra of the unknown compounds were compared with those of the known compounds stored in the NIST library. The name, molecular weight and structure of the compounds extracted from the test materials were ascertained.

## Results and discussion

### MTT assay

**Figure 1:**
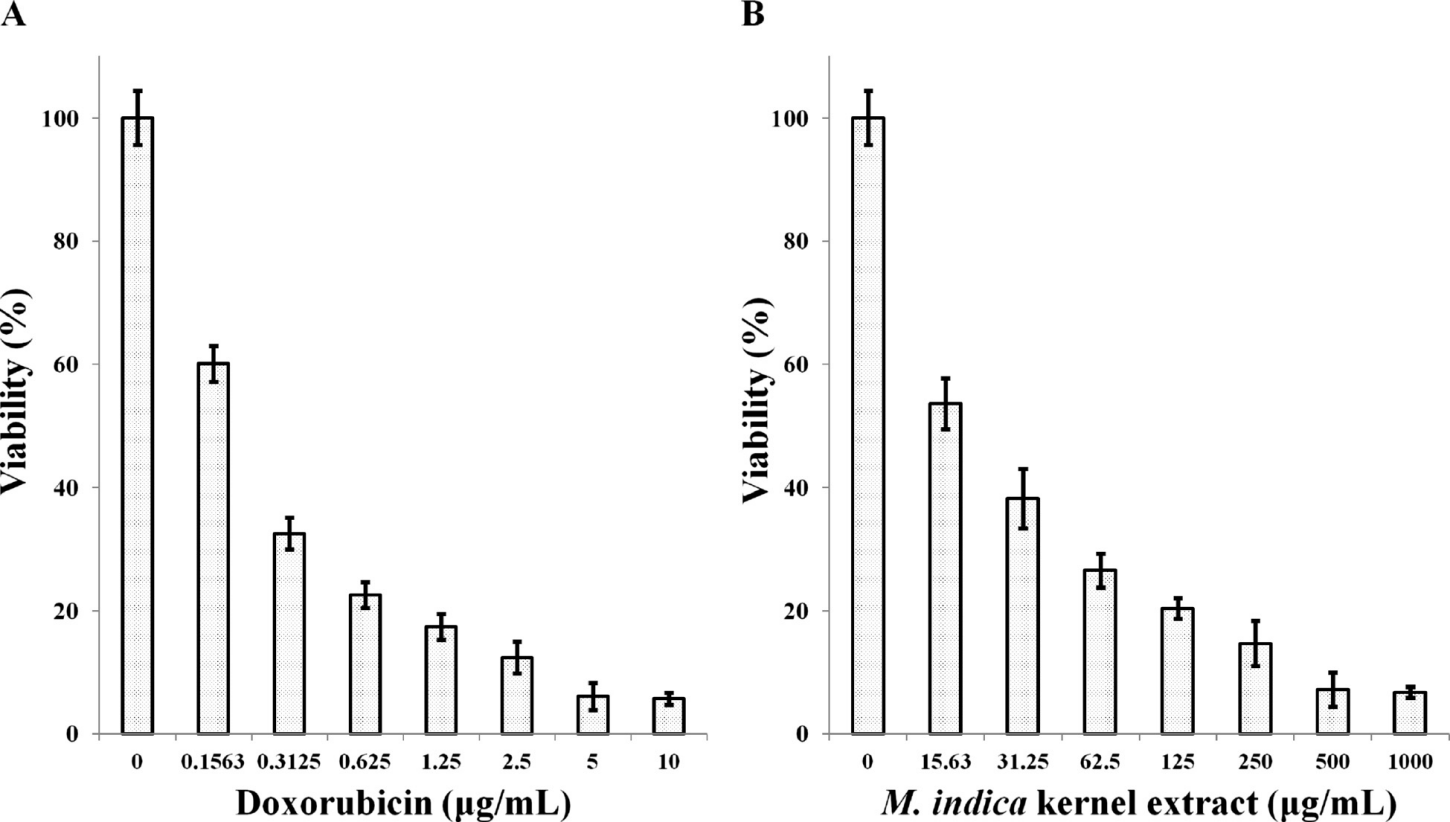
**Viability of MCF-7 cells after 72 hours treatment with (A) doxorubicin and, (B) *M. indica *kernel extract.** Values are expressed as mean ± std. dev (*n* = 3).

**Figure 2:**
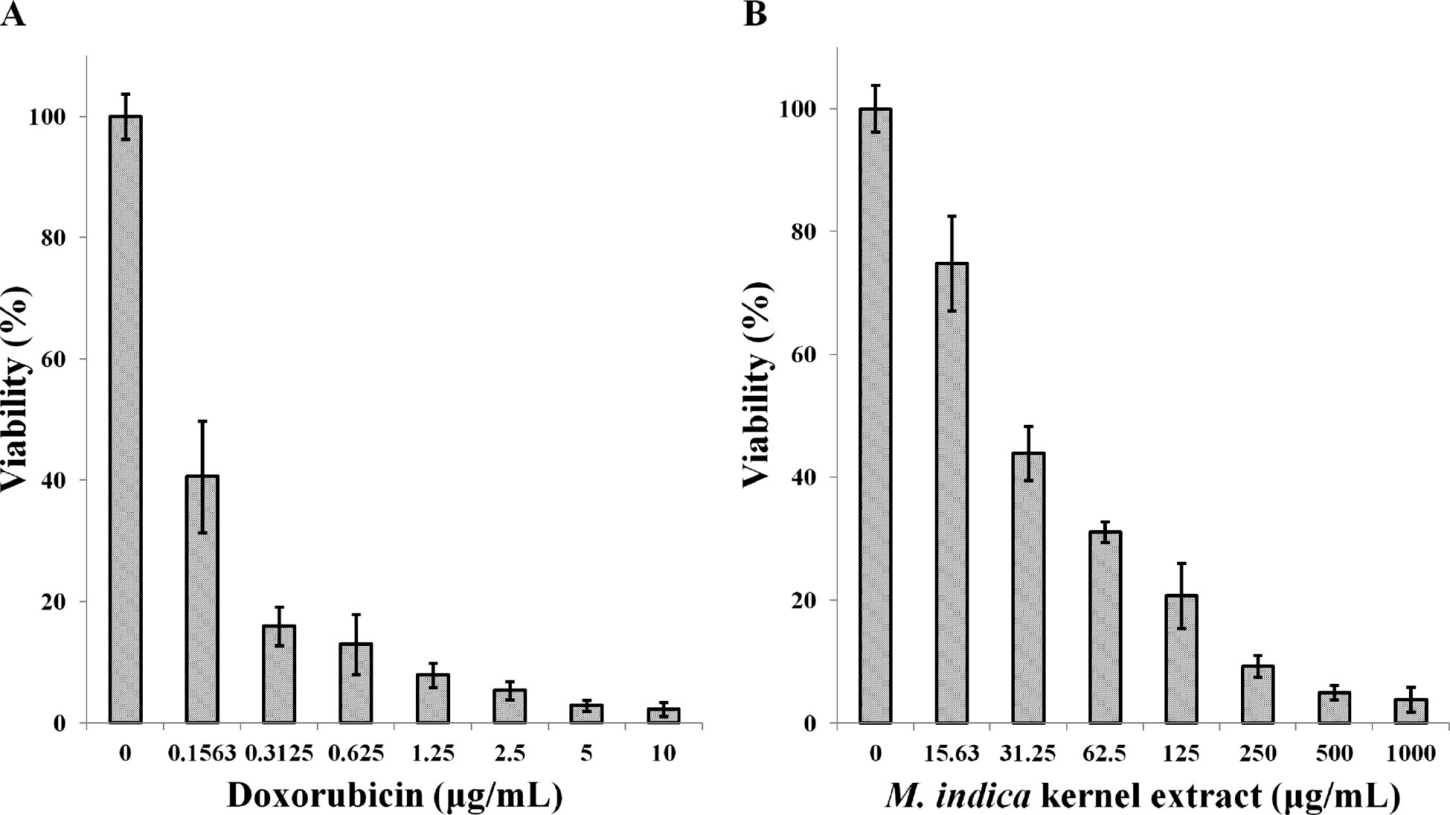
**Viability of MDA-MB-231 cells after 72 hour treatment with (A) doxorubicin and, (B) *M. indica *kernel extract.** Values are expressed as mean ± std. dev. (*n* = 3).

Two human breast carcinoma cell lines, estrogen receptor negative (ER-) MDA-MB-231 cells and estrogen receptor positive (ER+) MCF-7 cells were used to determine the cytotoxicity of the *M. indica* kernel extract against the cells. Non-tumorous MCF-10A cells were used as controls. The survival of the three human breast-derived cells after treatment with the kernel extract and doxorubicin after 72 h was determined. The responses of MCF-7 and MDA-MB-231 cancer cells to increasing concentrations of the kernel extract and doxorubicin are shown in Figures 1 and 2, respectively. The results showed the tendency of both cell lines to decrease sharply upon treatment with low extract and doxorubicin concentrations, with a tapering response intensity as the concentrations of the extract and doxorubicin were increased.

**Figure 3:**
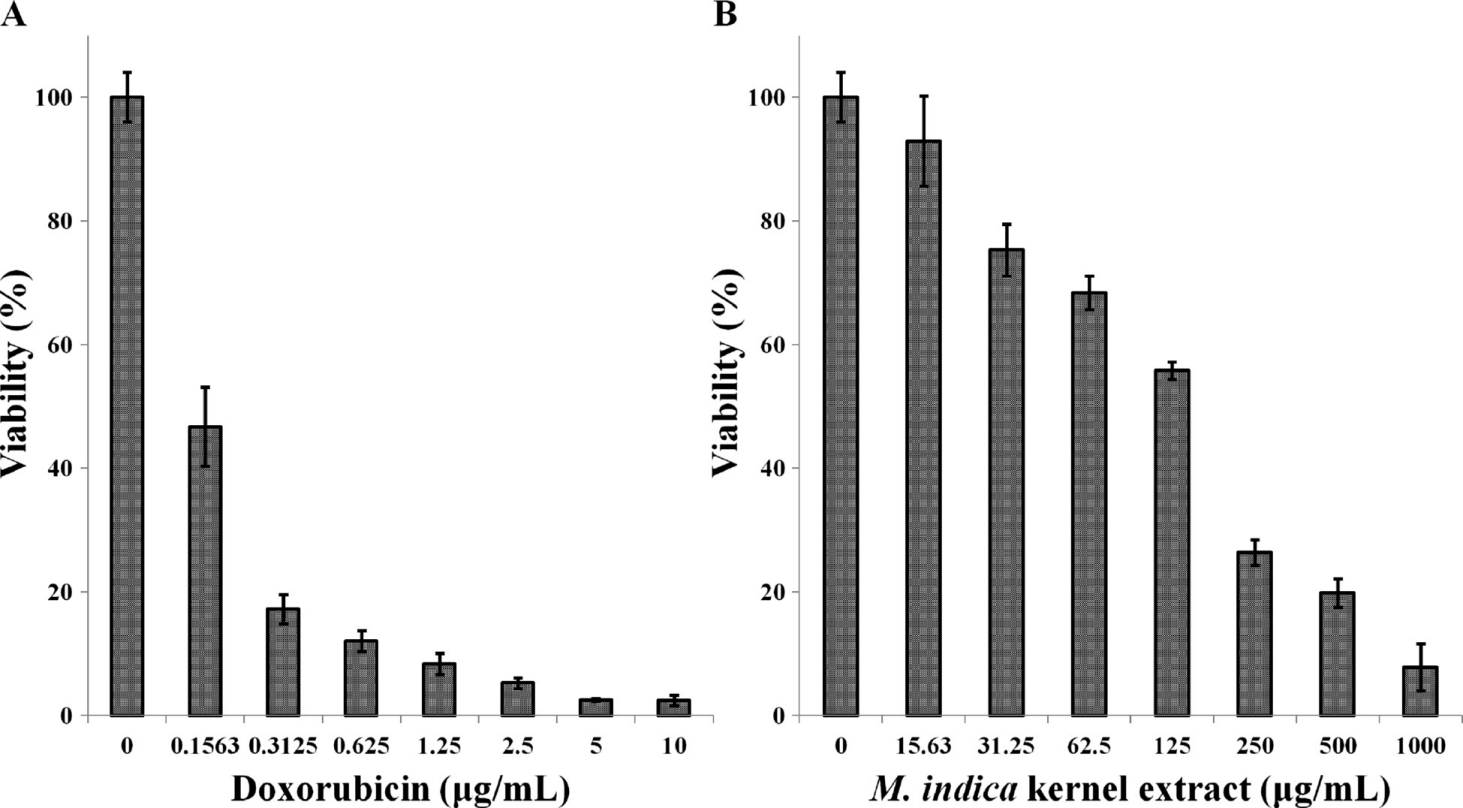
**Viability of MCF-10A cells after 72 hour treatment with (A) doxorubicin and, (B) *M. indica *kernel extract.** Values are expressed as mean ± std. dev. (*n* = 3).

**Figure 4:**
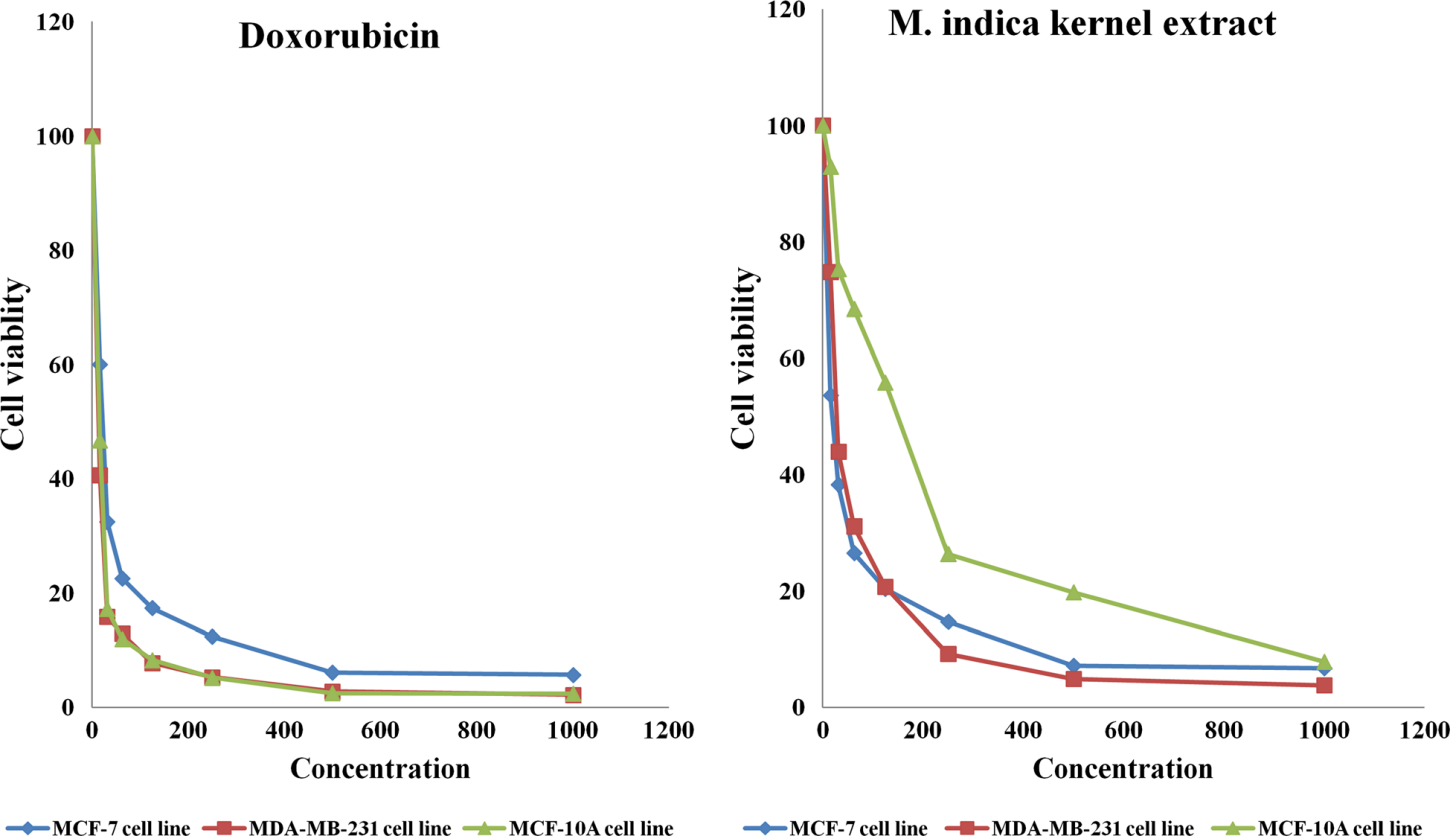
**Scatter plot showing viability of breast cancer cells treated with doxorubicin or *M. indica *kernel extract after 72 hours.** Values are expressed as mean ± std. dev (*n* = 3).

Following 72 h incubation with 15.6 μg/mL of the extract, MCF-7 and MDA-MB-231 cell retained 53 and 76% viability, respectively, indicating decrease in cell growth by 47 and 24% for the MCF-7 and MDA-MB-231 cell, respectively. The MCF-10A cells similarly treated with kernel extract showed only 7% decrease in growth thereby retaining 93% viability (Figure 3). This insignificant decrease in growth of MCF-10A 223 as compared to considerably higher values seen in MCF-7 and MDA-MB-231 indicates that the extract exhibited low toxicity towards normal cells and significantly higher toxicity towards the cancer cells. The IC_50_ values of the kernel extract after 72 h treatment ranged between 15 and 30 μg/mL for MCF-7 and MDA-MB-231 cell lines, while it was found to be significantly higher (149 μg/mL) for the normal MCF-10A cells (Figure 4). The kernel extract showed lower IC_50_ values for the cancer cells compared to the normal breast cell, suggesting that the extract could have huge potentials as an anti-cancer agent. These findings mirrored those of Abu Bakar *et al*. [23], who demonstrated the anti-cancer potentials of the seed kernel of *Mangifera pajang* (Banbangan) on the same type of cell lines following similar treatments.

### Anti-proliferation assay

**Figure 5:**
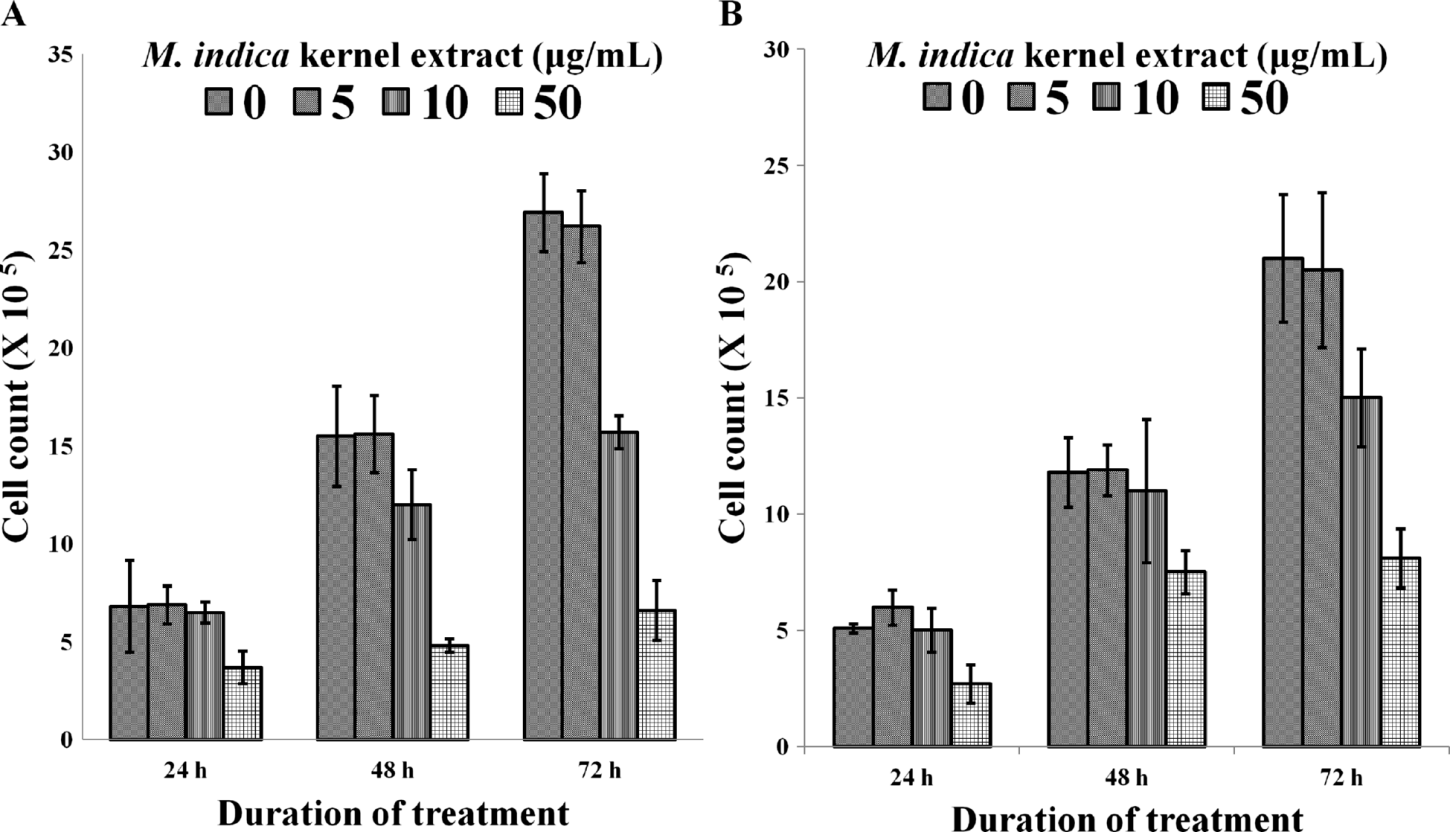
**Viability of breast cancer cells treated with *M. indica *kernel extract after 24**, **48 and 72 hours, (A) MCF-7 cell and (B) MDA-MB-231 cell.** Values are expressed as mean ± std. dev (*n* = 3).

Figure 5 illustrates the anti-proliferative effects of the kernel extract on MCF-7 and MDA-MB-231 cells. The percentage survival of the MCF-7 cells after 24, 48 and 72 h of incubation with 10 μg/mL of extract were 96, 77 and 52%, respectively. However, MDA-MB-231 cells similarly treated with the kernel extract did not show as much reduction in viability as the MCF-7 cells with values of 98, 93 and 71%, respectively. This indicated that the kernel extract could have greater effect on the viability of MCF-7 than MDA-MB-231 cells.

### NR uptake

**Figure 6:**
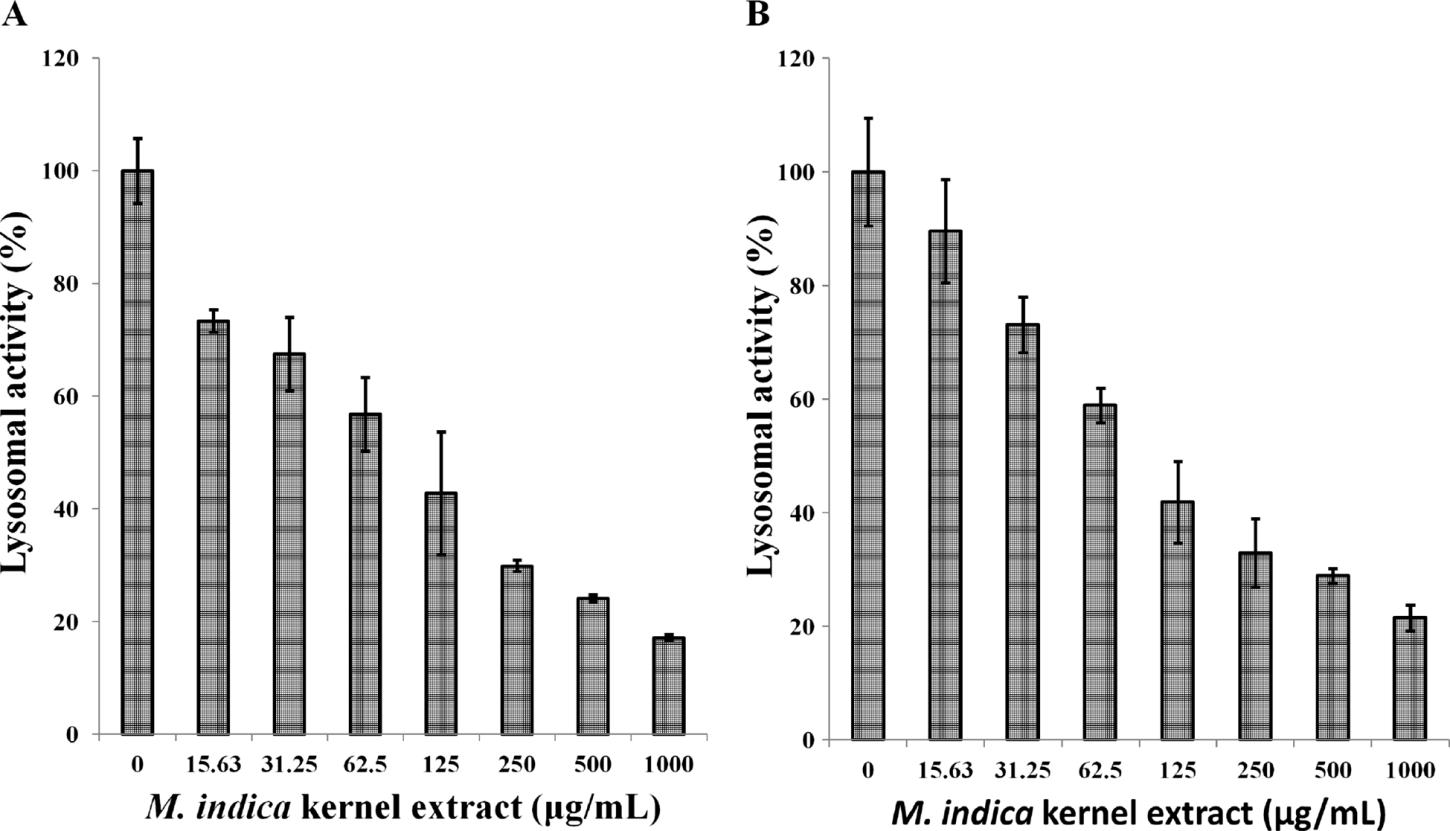
**Lysosomal activity of breast cancer cell lines treated with *M. indica *kernel extract for 72 hours, determined by neutral red uptake assay. (A)** MCF-7 cell line and, **(B)** MDA-MB-231 cell line. Values are expressed as mean ± std. dev. (*n* = 3).

The NR assay used to determine lysosomal activity of MCF-7 and MDA-MB-231 cells treated with the kernel extract showed lower sensitivity than the MTT assay although a significant decrease in lysosomal activity in a dose-dependent manner was observed (Figure 6). The lower sensitivity may be due to the lower numbers of lysosomes in the breast cancer cell lines. NR uptake assay is based on the capacity of viable cells to bind and incorporate the supravital dye, NR, into lysosomes. NR is a positively charged dye that passively diffuses across cellular membrane and accumulates in the lysosomes, and the intensity of its staining is directly proportional to the number of viable cells.

### LDH release assay

**Figure 7:**
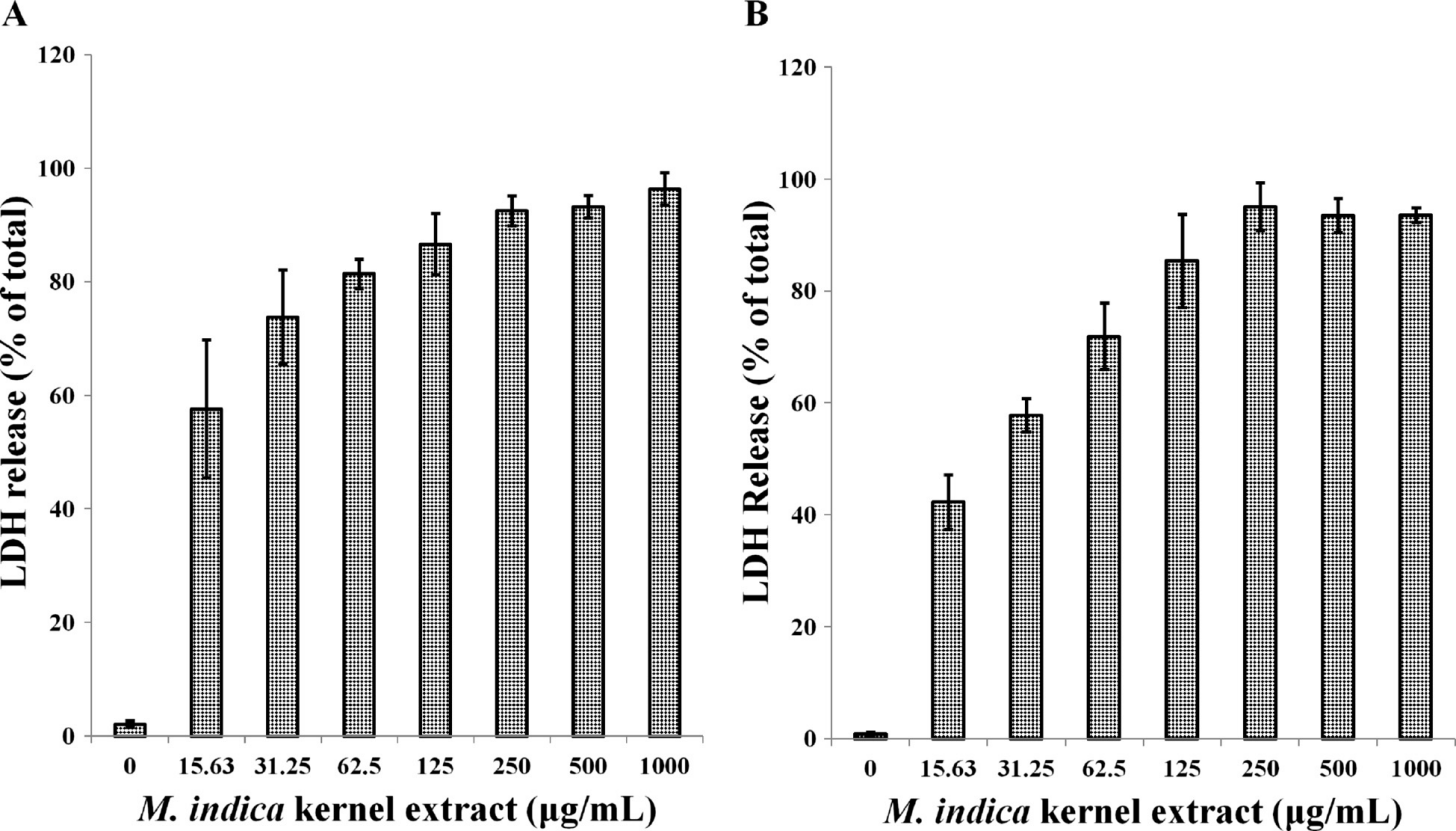
**Lactate Dehydrogenase (LDH) release from (A) MCF-7 cells and, (B) MDA-MB-231 cells treated with *M. indica *kernel extract.** The number of cells used for each treatment was 1 × 10^4^.

Cell death in culture was also determined by the release of LDH into the incubation medium. The LDH release curves for MCF-7 and MDA-MB-231 cell lines treated with different concentrations of the kernel extract suggested that the cytotoxic effect of the extract was concentration-dependent (Figure 7). The percentage of LDH release from MCF-7 cell lines after 72 h exposure to 15, 31, 62, and 125 μg/mL extract concentrations were 57, 73, 81 and 86%, respectively. This effect is greater than those observed for the same concentration of extract on MDA-MB-231 cell lines with 42, 58, 72 and 85%, respectively after 72 h. However, higher concentrations (250–1000 μg/mL) of the extract produced even greater LDH release on both cells.

### Analysis of bioactive compounds

**Figure 8:**
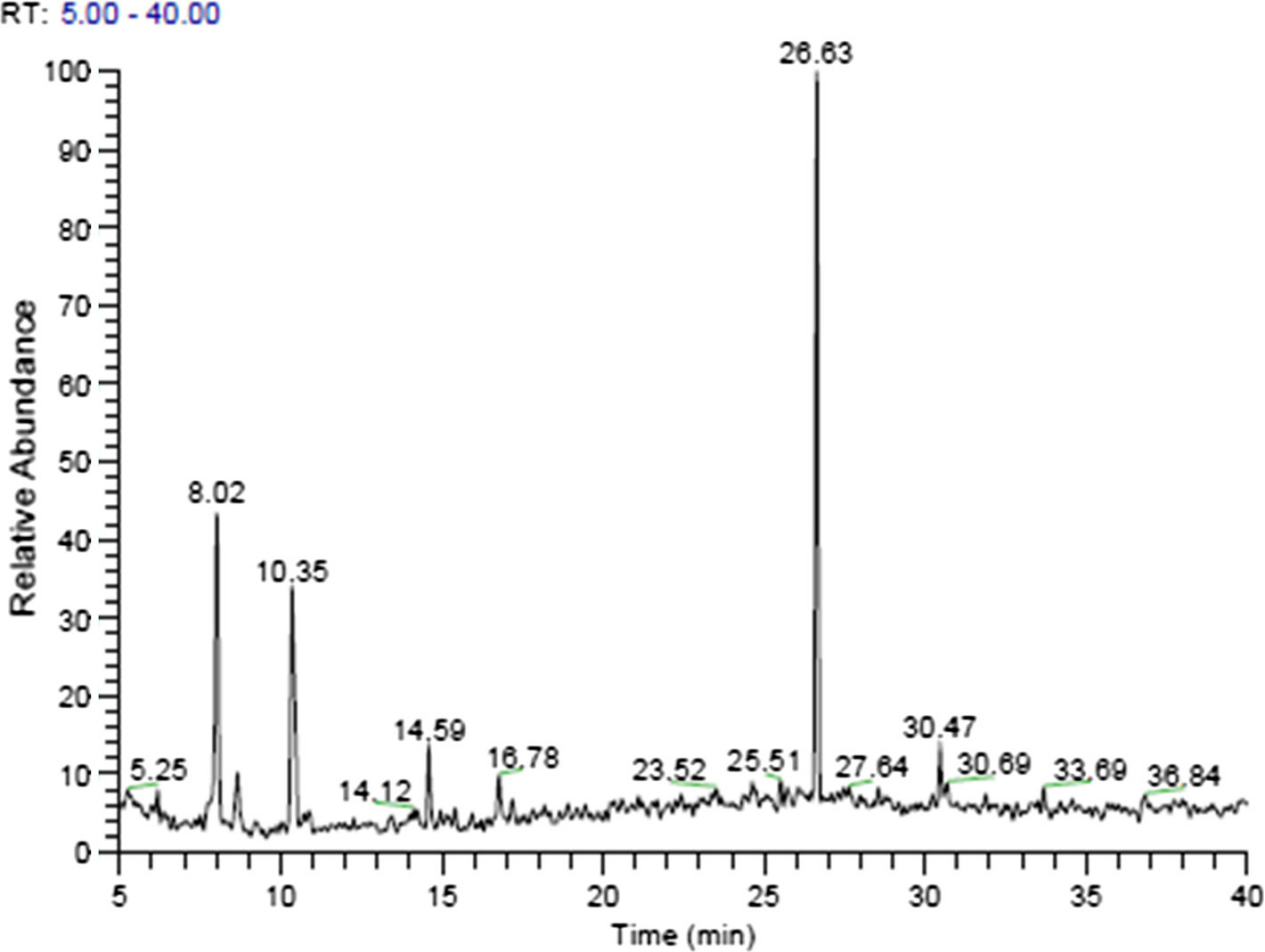
**GC-MS chromatogram of the ethanolic extract of the Mango (Waterlily) kernel extract, giving out twelve apparent compounds.**

Twelve major compounds were identified in the kernel extract of *Mangifera indica* L. The GC-MS chromatogram is shown in Figure 8, and the corresponding compounds with their retention times, molecular formulae, molecular weights (MW), and concentrations (%) are shown in Table 1.

**Table 1:** Common biological activities of the phytocomponents identified in the ethanolic extract of Waterlily kernel extract

**No.**	**Retention time (min)**	**Name of compound**	**Molecular formula and molecular weight (MW)**	**Peak area (%)**
1.	*8.02*	*1*-*Bu*tanol, 3-methyl-, acetate	C_7_H_14_O_2_	17.85
			MW: 130	
2.	10.35	Butane, 1,1-diethoxy-*3*-*methyl*-	*C*_*9*_*H*_*20*_*O*_*2*_	20.79
			MW: 160	
3.	14.59	Propane, 1,1,3-triethoxy-	C_9_H_20_O_3_	3.00
			MW: 176	
4.	16.77	Ethaneperoxoic acid, 1-cyano-1-(2-methylphenyl) ethyl ester	C_12_H_13_NO_3_	2.03
			MW: 219	
5.	23.54	Apigenin 7-glucoside	C_21_H_20_O_10_	1.74
			MW: 432	
6.	26.08	Disperse Red 11	C_15_H_12_N_2_O_3_	5.72
			MW: 268	
7.	26.63	Phenol, 4,6-di (1,1-dimethylethyl)-2-methyl- (Butylated Hydroxytoluene, BHT)	C_15_H_24_O	44.65
			MW: 220	
8.	30.47	Chlorazanil	C_9_H_8_ClN_5_	4.73
			MW: 221	
9.	30.69	Isoheptadecanol (1-Hexadecanol,2-methyl)	C_17_H_36_O	2.74
			MW: 256	
10.	30.82	cis-5-Dodecenoic acid, (3-cyanopropyl) dimethylsilyl ester	C_18_H_33_NO_2_Si	1.47
			MW: 323	
11.	32.78	Fumaric acid, 2-decyl undecyl ester	C_25_H_46_O_4_	2.85
			MW: 410	
12.	36.84	Phthalic acid, hept-2-yl isohexyl ester	C_21_H_32_O_4_	2.47
			MW: 348	

Of the twelve compounds detected, five compounds have been previously reported to possess anticancer potentials, including phenol, 4,6-di (1,1-dimethylethyl)-2-methyl- (44.65%) [24], Fumaric acid, 2-decyl undecyl ester (2.85%) [25], Isoheptadecanol (1-Hexadecanol, 2-methyl) (2.74%) [26], Apigenin 7-glucoside (1.74%) [27,28], and cis-5-Dodecenoic acid, (3-cyanopropyl) dimethylsilyl ester (1.47%) [29]. In addition, the other compounds have also been shown to exhibit some biological activities including antioxidant activity. Phenol, 4,6-di (1,1-dimethylethyl)-2-methyl- also known as butylated hydroxytoluene (BHT) was found to be the most abundant in the extract, and is a common food additive that is reported to have high antioxidant potentials. It is commonly used in the pharmaceutical, cosmeceutical and other industries largely due to its antioxidant properties. Apigenin 7-glucoside, a phenolic compound with potent antioxidant and anticancer potentials [30], was also found to be present in the extract.

It is most probable that the cytotoxic effects of the extract of *Mangifera indica* L. on breast cancer cells as shown in this study are due to more than one bioactive compound in view of the multiple compounds detected in the extract and since a crude extract was used. Already, some of the compounds found in the extract have been reported to have effects favorable for an anticancer activity, and these effects could have been synergistically contributed to the anticancer potentials observed in the extract.

## Conclusions

The MTT, anti-proliferation, NR uptake and LDH release assays used to evaluate the cytotoxicity of ethanolic kernel extract of *Mangifera indica* L on MCF-7 and MDA-MB-231 cell lines showed that the extract is significantly cytotoxic to these cell lines in a dose-dependent manner, and considerably less so towards normal breast cells MCF-10A. These findings highlights the potentials of *Mangifera indica* L extract in the treatment of breast cancer. Its therapeutic potential is huge and can be used as alternative to or supplementation for the various therapy currently used in the treatment of breast cancer. However there is a need to identify the actual components responsible for this cytotoxicity and to isolate them and to study their effect in vivo to ascertain their efficacies and or any side effects. The concerns of side effects with pharmacological agents, and growing interest in plant bioresources for treatment of cancers mean this extract could have important role in future studies on the mangement of breast cancer.

## Abbreviations

DMEM: Dulbecco’s modified Eagle medium; DMSO: Dimethylsulphoxide; ER-: Estrogen receptor negative; ER+: Estrogen receptor positive; FBS: Fetal bovine serum; GC-MS: Gas chromatography–mass spectrometry; LDH: Lactate dehydrogenase; MTT: Thiazoyl blue tetrazolium bromide; NADH: Nicotinamide adenine dinucleotide; NR: Neutral red; PBS: Phosphate buffer saline.

## Competing interest

The author’s declared that they have no cmpeting interests.

## Author’s contributions

AHA designed the study and carried out most of the experimental parts, while MA contributed to the cell culture assays. ASM, RA and MESM contributed towards supervision of the work. They also read, revised and approved the final manuscript for submission critically. All authors read and approved the final manuscript.

## Pre-publication history

The pre-publication history for this paper can be accessed here:

http://www.biomedcentral.com/1472-6882/14/199/prepub

## References

[B1] Angelopoulos N Barbounis V Livadas S Kaltsas D Tolis G Effects of estrogen deprivation due to breast cancer treatment Endocr Relat Cancer 2004 11 523 535 10.1677/erc.1.00783 15369452

[B2] Jemal A Siegel R Ward E Murray T Xu J Smigal C Thun MJ Cancer Statistics CA Cancer J Clin 2006 56 106 130 10.3322/canjclin.56.2.106 16514137

[B3] Ferguson PJ Kurowska E Freeman DJ Chambers AF Koropatnick DJ A flavonoid fraction from cranberry extract inhibits proliferation of human tumor cell lines J Nutr 2004 134 1529 1535 15173424 10.1093/jn/134.6.1529

[B4] Jo EH Hong HD Ahn NC Jung JW Yang SR Park JS Kim SH Lee YS Kang KS Modulations of the Bcl-2/Bax family were involved in the chemopreventive effects of licorice root (Glycyrrhiza uralensis Fisch) in MCF-7 human breast cancer cell J Agric Food Chem 2004 52 1715 1719 10.1021/jf035012t 15030235

[B5] Mukherjee AK Basu S Sarkar N Ghosh AC Advances in cancer therapy with plant based natural products Curr Med Chem 2001 8 1467 1486 10.2174/0929867013372094 11562277

[B6] Chen MS Chen D Dou QP Inhibition of proteasome activity by various fruits and vegetables is associated with cancer cell death In Vivo 2004 18 73 80 15011755

[B7] Cragg GM Newman DJ Plants as a source of anti-cancer agents J Ethnopharmacol 2005 100 72 79 10.1016/j.jep.2005.05.011 16009521

[B8] Tan G Gyllenhaal C Soejarto DD Biodiversity as a source of anticancer drugs Curr Drug Targets 2006 7 265 277 10.2174/138945006776054942 16515527

[B9] Ivanova D Gerova D Chervenkov T Yankov T Polyphenols and antioxidant capacity of Bulgarian medicinal plants J Ethnopharmacol 2005 96 145 150 10.1016/j.jep.2004.08.033 15588663

[B10] Fleischauer AT Simonsen N Arab L Antioxidant supplements and risk of breast cancer recurrence and breast cancer-related mortality among postmenopausal women Nutr Cancer 2003 46 15 22 10.1207/S15327914NC4601_02 12925299

[B11] Aziz MH Kumae R Ahmad N Cancer chemoprevention by resveratrol: In vitro and in vivo studies and the underlying mechanisms Int J Oncol 2003 23 17 28 Review 12792772

[B12] Primchanien M Nuttavut K Sineenart K Omboon L Narongchai P Neelobol N Antiproliferation, antioxidation and induction of apoptosis by Garcinia mangostana (mangosteen) on SKBR3 human breast cancer cell line J Ethnopharmacol 2004 92 161 166 10.1016/j.jep.2003.09.048 14698525

[B13] Naasani I Oh-Hashi F Oh-Hara T Feng WY Johnston J Chan K Tsuruo T Blocking telomerase by dietary polyphenols is a major mechanism for limiting the growth of human cancer cells in vitro and in vivo Cancer Res 2003 63 824 830 12591733

[B14] Cai Y Luo Q Sun M Corke H Antioxidant activity and phenolic compounds of 112 traditional Chinese medicinal plants associated with anticancer Life Sci 2004 74 2157 2184 10.1016/j.lfs.2003.09.047 14969719 PMC7126989

[B15] Kim Y Brecht JK Talcott ST Antioxidant phytochemical and fruit quality changes in mango (*Mangifera indica L*.) following hot water immersion and controlled atmosphere storage Food Chem 2007 105 1327 1334 10.1016/j.foodchem.2007.03.050

[B16] Krishna H Singh SK Biotechnological advances in mango (*Mangifera indica L*.) and their future implication in crop improvement: a review Biotech Adv 2007 25 223 243 Review 10.1016/j.biotechadv.2007.01.001 17321096

[B17] Robles-Sanchez RM Rojas-Grau MA Odriozola-Serrano I Gonzalez-Aguilar GA Martin-Belloso O Effect of minimal processing on bioactive compounds and antioxidant activity of fresh-cut ‘Kent’ mango (*Mangifera indica L*.) Postharvest Biology and Tech 2009 51 384 390 10.1016/j.postharvbio.2008.09.003

[B18] Ling LT Yap SA Radhakrishnan AK Subramaniam T Cheng HM Palanisamy UD Standardised Mangifera indica extract is an ideal antioxidant Food Chem 2009 113 1154 1159 10.1016/j.foodchem.2008.09.004

[B19] Kim H Moon JY Kim H Lee D-S Cho M Choi H-K Kim YS Mosaddik A Cho SK Antioxidant and antiproliferative activities of mango (*Mangifera indica* L.) flesh and peel Food Chem 2010 121 429 436 10.1016/j.foodchem.2009.12.060

[B20] Suhr KI Nielsen PV Antifungal activity of essential oils evaluated by two different application techniques against rye bread spoilage fungi J Appl Microbiol 2003 94 665 674 10.1046/j.1365-2672.2003.01896.x 12631202

[B21] Al-Qubaisi M Rozita R Yeap S-K Omar A-R Ali A-M Alitheen NB Selective cytoxicity of goniothalamin against hepatoblastoma HepG2 cells Molecules 2011 16 2944 2959 10.3390/molecules16042944 21471934 PMC6260619

[B22] Hema R Kumaravel S Gomathi S Sivasubramaniam C Gas chromatography-Mass Spectroscopic analysis of *Lawsonia inermis* leaves New York Sci J 2010 3 141 143

[B23] Abu Bakar MF Mohamad M Rahmat A Burr SA Fry JR Cytoxicity, cell cycle arrest and apoptosis in breast cancer cell lines exposed to an extract of the seed kernel of *Mangifera pajang* (bambangan) Food Chem Toxicol 2010 48 1688 1697 10.1016/j.fct.2010.03.046 20363279

[B24] Hocman G Chemoprevention of cancer: phenolic antioxidants (BHT, BHA) Int J Biochem 1988 20 639 651 10.1016/0020-711X(88)90158-9 3053283

[B25] Kuroda K Teraro K Akao M Inhibitory effect of fumaric acid on hepatocarcinogenesis by thioacetamide in rats J Natl Cancer Inst 1987 79 1047 1051 3479633

[B26] Okokon JE Dar A Choudhary MI Immunomodulatory, cytotoxic and antileishmanial activity of phytoconstituents of *Croton zambesicus* Phytopharmacol 2013 4 31 40

[B27] El-Alfy TS El-Sawi SA Sleem A Moawad DM Investigation of Flavonoidal Content and biological activities of *Chorisia Insignis* Hbk. leaves Austr J Basic Appl Sci 2010 4 1334 1348

[B28] Gulluce M Orhan F Yanmis D Arasoglu T Guvenalp Z Dermirezer LO Isolation of a flavonoid, apigenin 7-O-glucoside, from *Mentha longifolia* (L.) Hudson subspecies longifolia and its genotoxic potency Toxicol Ind Health 2013 doi:10.1177/0748233713475511 10.1177/0748233713475511 23377117

[B29] Posner GH Ploypradith P Hapangama W Wang D Cumming JN Dolan P Kensler TW Klinedinst D Shapiro TA Zheng QY Murray CK Pilkington LG Jayasinghe LR Bray JF Daughenbaugh R Trioxane dimers have potent antimalarial, antiproliferative and antitumor activities in vitro Bioorgan Med Chem 1997 5 1257 1265 10.1016/S0968-0896(97)00079-5 9377085

[B30] Nakazaki E Tsolmon S Han J Isoda H Proteomic study of granulocytic differentiation induced by apigenin 7-glucoside in human promyelocytic leukemia HL-60 cells Eur J Nutr 2013 52 25 35 10.1007/s00394-011-0282-4 22113421

